# Vaccination with recombinant *Toxoplasma gondii* bradyzoite-formation deficient 1 (rTgBFD1) antigen provides partial protective immunity against chronic *T. gondii* infection

**DOI:** 10.3389/fvets.2022.957479

**Published:** 2022-09-12

**Authors:** Xiaowei Tian, Zhenke Yang, Guangmin Wan, Tong Xie, Meng Wang, Hanqi Sun, Xuefang Mei, Zhenchao Zhang, Xiangrui Li, Shuai Wang

**Affiliations:** ^1^Xinxiang Key Laboratory of Pathogenic Biology, Department of Pathogenic Biology, School of Basic Medical Sciences, Xinxiang Medical University, Xinxiang, China; ^2^MOE Joint International Research Laboratory of Animal Health and Food Safety, College of Veterinary Medicine, Nanjing Agricultural University, Nanjing, China

**Keywords:** *Toxoplasma gondii*, bradyzoite-formation deficient 1, recombinant antigen, protective immunity, BALB/c mice

## Abstract

As an apicomplexan pathogen, *Toxoplasma gondii* still remains a major threat to public health and requires special attention. In fact, positive attempts to identify more effective antigens to provide protection are important to control toxoplasmosis. Latest scientific advances in *T. gondii* study hint at the probability of the *T. gondii* bradyzoite-formation deficient 1 (TgBFD1) as an ideal vaccine candidate, since this molecule plays a critical role in regulating the chronic infection of *T. gondii*. Thus, BALB/c mouse models of acute and chronic *T. gondii* infections were used to evaluate the TgBFD1 protection efficacy in this study. Before conducting animal trials, antigen analysis of TgBFD1 was performed using DNAstar software and Western blots. The preliminary results suggested that TgBFD1 should be a potent immunogen. Then, this conclusion is confirmed by ELISA assays. After immunization with rTgBFD1, high levels of specific IgG, IgG1, IgG2a, and cytokines (Interferon γ and interleukin 10) were observed, indicating that TgBFD1 could induce strong protective antibody responses. While TgBFD1-specific IgG antibodies were measurable in vaccinated mice, no protection was observed in the acute *T. gondii* infection (RH strain) assay. However, a noticeable decrease in brain cysts counts of immunized mice compared with negative controls in the latent *T. gondii* infection (PRU strain) assay was observed. Taken together, these results indicated that rTgBFD1 had the remarkable ability to elicit both humoral and cellular immune responses and could provide partial protective immunity against chronic *T. gondii* infection.

## Introduction

*Toxoplasma gondii* (*T. gondii*) is an opportunistic protozoon that is known to be responsible for approximately one-third of the global population infection (1, 2). There is a risk of infantile encephalitis, miscarriage in pregnant women and livestock, and even death for immunocompromised individuals due to this disorder, which poses serious harm to the public and animal health (3–5). Tachyzoites rapidly invade host-nucleated cells during the initial *T. gondii* infection. It is possible for tachyzoites to turn into bradyzoites for developing latent tissue cysts, which can lead to chronic infection in immunocompetent hosts (6).

Until present, toxoplasmosis cannot be fully cured by currently available pharmacotherapy. Thus, immunoprophylaxis is the preferred method for controlling the disease (7–10). However, so far, no vaccine is available for preventing human toxoplasmosis. Over past decades, several vaccine forms, such as inactivated vaccines, live attenuated vaccines, DNA vaccines, and subunit vaccines, are considered to make safe and efficient vaccines suitable for humans (11). There is only one licensed vaccine against *T. gondii* at present: Toxovax (12). However, this vaccine cannot be applied for humans, because its format may revert to virulence. Therefore, discovering safe and effective antigens is urgent.

In recent years, multiple attempts have been made to identify the protective antigens from *T. gondii*. The antigens such as rhoptry proteins, dense granule proteins, and surface antigens expressed in tachyzoite and/or bradyzoite stages have exhibited the tremendous potential to be vaccine candidates (13, 14), and of these, proteins expressed in the bradyzoite stage deserve more attention because bradyzoites have long-term latent infection ability. Studies demonstrated that bradyzoite-specific antigens BAG1 and MAG1 could induce early humoral and cell-mediated immune responses upon human infection (15). Nevertheless, the research focused on the bradyzoite proteins was still limited.

A recent study suggested that bradyzoite-formation deficient 1 (TgBFD1) can be used as the candidate for the vaccine. Waldman et al. found TgBFD1 was a master regulator of chronic phase. *T. gondii* accumulates higher levels of TgBFD1 when faced with stress conditions, resulting in the conversion of the tachyzoite stage into bradyzoites. In addition, tachyzoites, which lack TgBFD1, could not transform into bradyzoites. Moreover, TgBFD1 played critical roles during cyst formation. TgBFD1-deficient strain can hardly generate tissue cysts in mice (16).

Current studies suggest that TgBFD1 may serve as a potential vaccine candidate for the control of *T. gondii*. The possible functions of TgBFD1 in the clearance of cysts provide reasons to expect its ability in providing immune protection against *T. gondii* infection. Taking into account that recombinant proteins for the production of vaccines are the appropriate method on a commercial basis (17), rTgBFD1 was performed for active immunizations. In this study, ISA 201 adjuvant (Seppic, France) is the appropriate choice considering the obvious advantages of its high safety profile, long-term protective immune response, and convenient-to-inject property (18). Therefore, we aimed to determine the protective efficacy of TgBFD1 in BALB/c mouse models infected with *T. gondii*. Furthermore, active immunized mice assays suggested that TgBFD1 could provide partial protection against infection caused by *T. gondii* RH or PRU strains. In comparison, TgBFD1 protection efficacy for chronic infection performed better.

## Materials and methods

### Mice and *T. gondii* strains

Female BALB/c mice (age 6 weeks) were bought from Charles River Animal Technology Company, Beijing, China and maintained under standard, pathogen-free conditions. Animal assays conducted in this study were reviewed and approved by the Ethics Committee of Xinxiang Medical University (No. XYLL-2021S016).

*T. gondii* strains (RH and PRU) were preserved in Xinxiang Key Laboratory of Pathogenic Biology, Department of Pathogenic Biology of Xinxiang Medical University in Henan, China. The tachyzoites of the *T. gondii* RH strain and the cysts of the *T. gondii* PRU strain were purified and collected as previously described (19).

### Sequence analysis

According to the sequence analysis of the TgBFD1 open reading frame obtained from the ToxoDB database (Accession number: TGME49_200385-t26_1), the feature key domain of TgBFD1 (921–1023aa) predicted by uniport was chosen for subsequent assays. In addition, the DNASTAR software (Madison, WI, United States) was carried out to evaluate the possibility of TgBFD1 feature key domain as a candidate vaccine antigen in terms of the surface probability, the antigenic index, the hydrophilic plot, and the flexible region.

### Recombinant TgBFD1 (rTgBFD1) antigen production

The sequence (921–1023aa) of TgBFD1 was successively cloned and inserted into a prokaryotic expression vector pET-30a. The technique of homologous recombination was performed for the construction of pET-30a-TgBFD1 using the ClonExpress II One Step Cloning Kit (Vazyme, Nanjing, China). Primer sequences were as follows: forward primer: taagaaggagatatacatatgATGTGGAGCGCTGAGGAGG (*Nde* I) and reverse primer: ctcgagtgcggccgcaagcttTCATAGGCGGGCATTGCTG (*Hind* III). rTgBFD1 was obtained by using *E. coli* BL-21 (DE3) competent cells and purified by the His Bind^®^ Resin Chromatography kit (Merck, Darmstadt, Germany). The purified rTgBFD1 was monitored by reducing SDS-PAGE. The endotoxin was depleted by the Detoxi-Gel Affinity Pak Prepacked columns (Pierce, Rockford, USA) as noted previously (20), and the rTgBFD1 was confirmed to be endotoxin free using the Gel Clot Limulus Amebocyte Lysate Single Test kit (BIOENDO, Xiamen China). The concentration of rTgBFD1 was determined using a BCA Protein Assay Kit (Boster Biological Technology, Wuhan, China). At last, the purified and endotoxin-free rTgBFD1 was stocked at −70°C for spare.

### Immunogenicity analysis of rTgBFD1

This immunogenicity analysis referred to the previous study (21). The rTgBFD1 was separated by 12% SDS-PAGE gel and transferred to the nitrocellulose membranes (Millipore, Shanghai, China). The blots were blocked with 5% skim milk in phosphate-buffered saline (PBS) with 0.5% Tween 20 (PBST) for 1 h at 37°C and then incubated with the serum collected from normal or *T. gondii*-infected mice (1:100 dilution) for 1 h at 37°C. After three washes, 5 min each using PBST buffer, the blots were incubated with goat anti-mouse IgG-HRP antibody (Sigma, Shanghai, China) (1:5000 dilution) for 1 h at 37°C. After three washes, 5 min each using PBST buffer, the blots were finally visualized by the enhanced chemiluminescence kit (Vazyme, Nanjing, China).

### Mice active immunization and infection assays

The 6-week-old BALB/c mice were randomly grouped (30 per group), and each mouse per group was immunized (three subcutaneous immunizations at 2-week intervals) with 20 μg of rTgBFD1 in 50 μl PBS emulsified with ISA 201 (volume 1:1), ISA 201 adjuvant (50 μl ISA 201 + 50 μl PBS), or PBS (100 μl PBS) alone. The day of immunization was set as week 0. To detect antibody levels and cytokine contents, the serum of immunized mice was collected on weeks 0 and 6, respectively, and then stored at −20°C for further use (*n* = 10). For *T. gondii* challenge assays, ten mice from each group were infected by intraperitoneal vaccination with 1 × 10^2^ RH strain tachyzoites, and the remaining ten mice were infected *via* oral gavage with 10 PRU strain cysts at 2 weeks post the last inoculation. The day of parasite infection was considered day 0. The survival of mice challenged by tachyzoites of *T. gondii* RH strain was recorded every 12 h until all mice had died. Mice infected by the *T. gondii* PRU cysts were observed daily for survival until 60 days after infection, and then, the cysts were removed from the brains for counting, which was conducted three times with a 10-μl brain mixture. The brain mixture was obtained by grinding with 1 ml PBS. The calculation of the cysts number reduction ratio was indicated as follows: cysts number of PBS control mice–adjuvant vaccinated mice or PBS control mice × 100%.

### Determination of antibody response in sera by ELISA

Based on previous literature (22), rTgBFD1-specific antibody levels in immunized mice serum were determined using ELISA with rTgBFD1. First, 5 μg/ml rTgBFD1 was used to cover the ELISA plates at 4°C overnight for examining specific IgG, IgG1, and IgG2a. Approximately 5% skim milk in PBST was used to block for 1 h at 37°C. After this operation, the ELISA plates were reacted with the primary antibody (immunized mice serum, 1:100 dilution) for 1 h at 37°C and subsequently reacted with the secondary antibodies (anti-mice IgG-HRP, IgG1-HRP, or IgG2a-HRP, 1:8000 dilution) for 1 h at 37°C. For specific IgG titers analysis, the primary antibody (immunized mice serum) was proceeded *via* serial dilution (1:10~1:10^5^). The ELISA plates washed with PBST were necessary after antibody incubations. Finally, after interaction with 3,3,5,5-tetramethylbenzidine, termination buffer 2 M H_2_SO_4_ was supplied to stop this reaction. Optical density at 450 nm (OD450) was performed using a microplate reader (Thermo Fisher Scientific, MA, United States).

### Cytokine detection

The mice sera obtained as described in the preceding paragraphs were used for the assessment of the cytokine content. In this assay, the commercial ELISA kits (Boster, Wuhan, China) were applied to quantitate cytokines interferon γ (IFN-γ), interleukin 4 (IL-4), interleukin 12p70 (IL-12p70), and interleukin 10 (IL-10) according to the manufacturer's instructions. Cytokine contents were figured out with the standard reference curve fitted by the mice recombinant IFN-γ, IL-12p70, IL-4, and IL-10 with a known quantity and their corresponding OD450. Four-parameter logistic regression equations were selected to fit these data. The units of four cytokine concentrations were picograms per milliliter (pg/ml).

### Statistical analysis

Data were statistically analyzed with the Graphpad Premier 8.0 software package (Graphpad Prism, CA, USA) in this study. Comparisons of the mean value (e.g., antibodies response, cytokines level) between all groups were conducted using one-way ANOVA. Survival analysis of *T. gondii*-infected mice was carried out by the Kaplan–Meier method. Comparisons were deemed significant between groups if the *p*-value was less than 0.05. For all figures, the following notations were set to denote significance: ^*^p < 0.05, ^***^p < 0.001, ns: non-significant.

## Results

### Secondary structure predictions of TgBFD1

**Figure 1:**
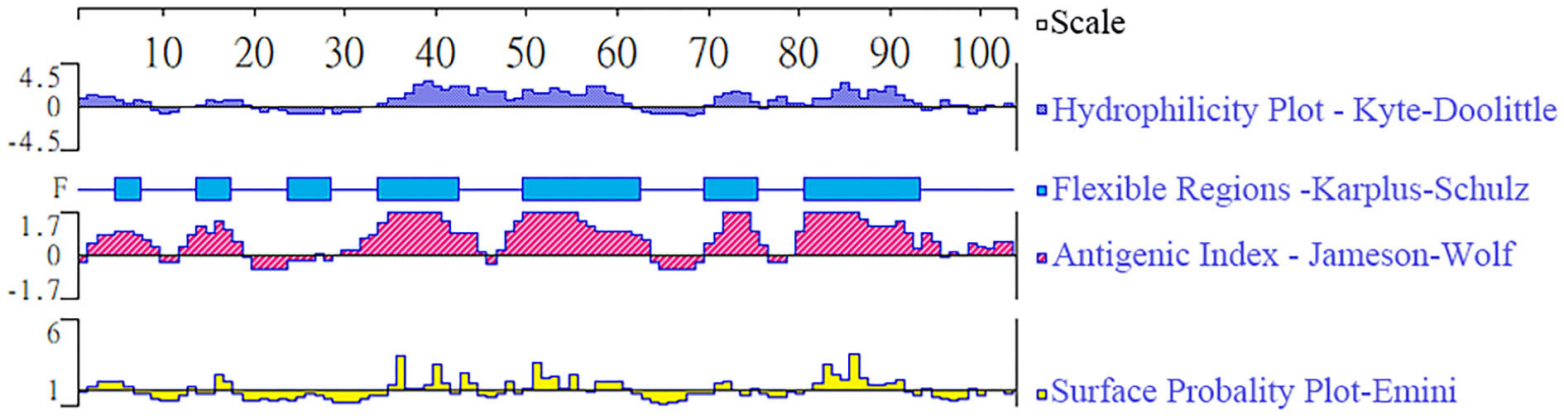
Epitope analysis of TgBFD1. The linear-B cell epitopes of the TgBFD1 feature key domain were analyzed by DNAstar software, such as hydrophilicity plot, flexible regions, antigenic index, and surface probability.

The epitope analysis is indicated in Figure 1, and the predicted region of TgBFD1 protein had predominant antigenic indices, which suggested that immunization with this antigen may provide a promising immune response.

### Cloning, expression, and purification of TgBFD1

**Figure 2:**
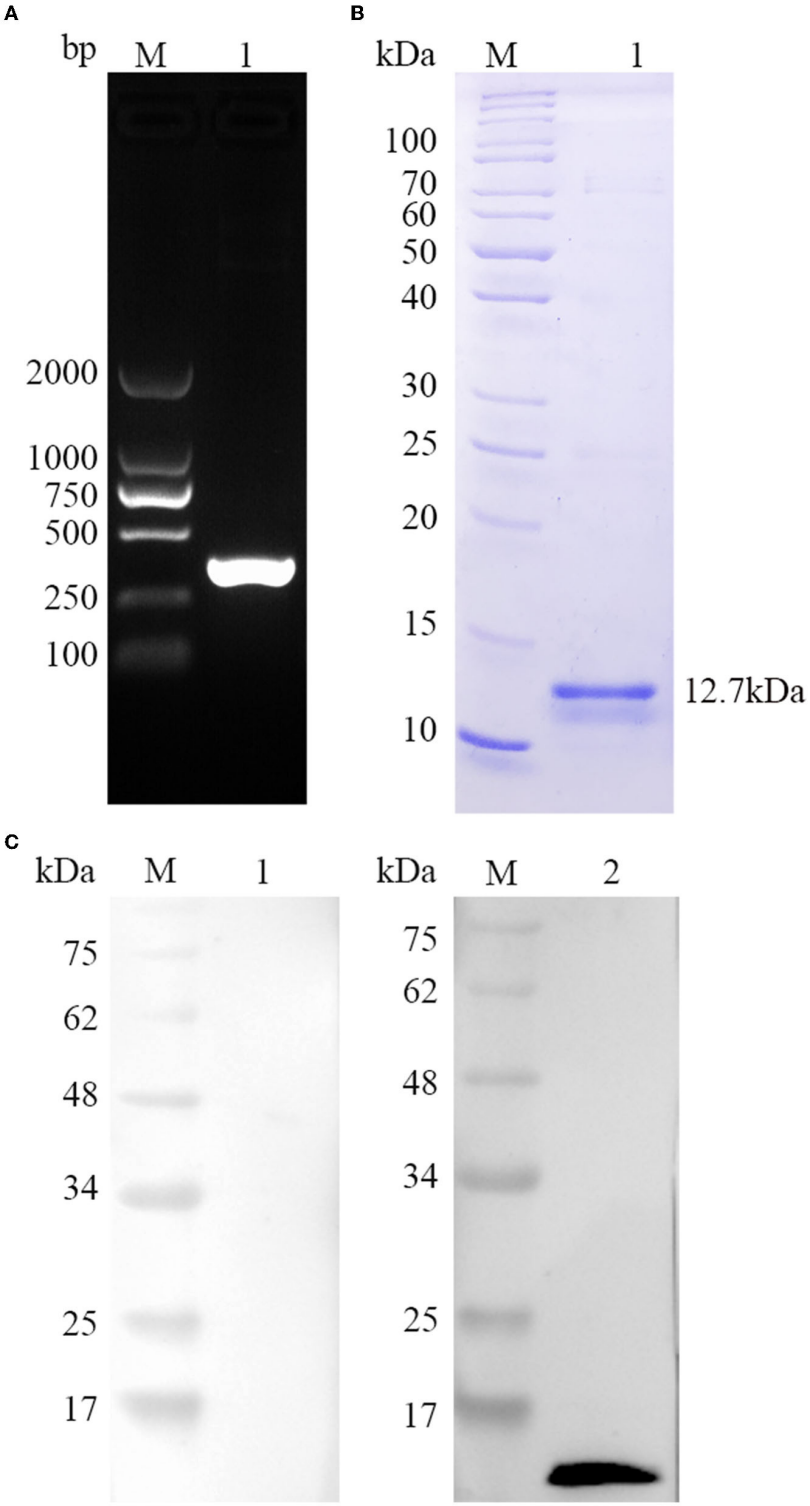
Gene amplification, protein purification, and recognition of TgBFD1. **(A)** The amplification of TgBFD1 was determined by 1% agarose gel electrophoresis. **(B)** Purified rTgBFD1 was run in 12% SDS-PAGE gel and visualized using Coomassie blue G250. **(C)** Recognition of rTgBFD1 was performed using toxoplasmosis mice sera. M, protein molecular mass standards; Line 1, normal mice IgG served as control; Line 2, sera obtained at 6 weeks after infection from mice infected with the *T. gondii* PRU strain served as a primary antibody.

As seen in Figure 2A, a single band size amplified by PCR was consistent with the TgBFD1 gene using 1% agarose gel. Then, the target sequence was extracted, cloned into prokaryotic expression vector pET-30a, and DNA sequencing was performed. To yield pure rTgBFD1, BL21(DE3) strain was chosen to transform reconstructed pET-30a-TgBFD1 plasmid, and nickel columns were performed to purify the His fusion rTgBFD1 using affinity chromatography. The purified rTgBFD1 was confirmed by 12% SDS-PAGE gel, which presented a single band at a 12.7-kDa level stained with Coomassie blue (Figure 2B).

### Immunogenicity of TgBFD1

The immunogenicity of TgBFD1 was evaluated to identify the vaccine viability of TgBFD1. As suggested in Figure 2C, a clear band around 12.7 kDa was typically detected in the positive control, whereas there was no band in the negative control using normal mice sera. The Western blot analysis of rTgBFD1 suggested that rTgBFD1 could be recognized by sera from *T. gondii*-infected mice.

### rTgBFD1 immunization could elicit a strong humoral immunological response

**Figure 3:**
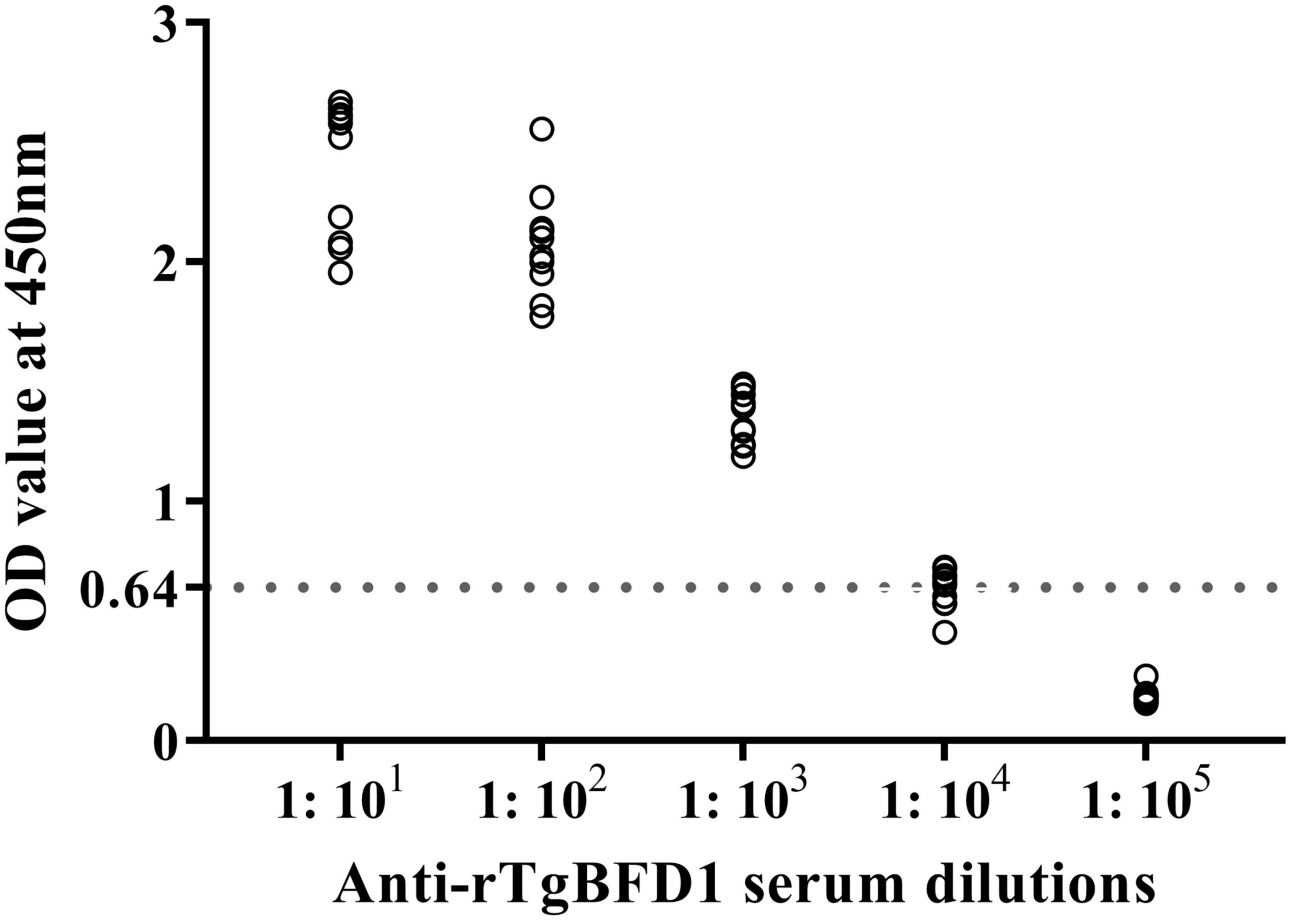
Antibody titers of rTgBFD1 specific IgG antibodies detected by rTgBFD1-ELISA. Ten rTgBFD1 immunized mice sera were collected and used for rTgBFD1-ELISA while the normal mice sera served as the negative control. The dotted line represents the cutoff value at 0.64.

**Figure 4:**
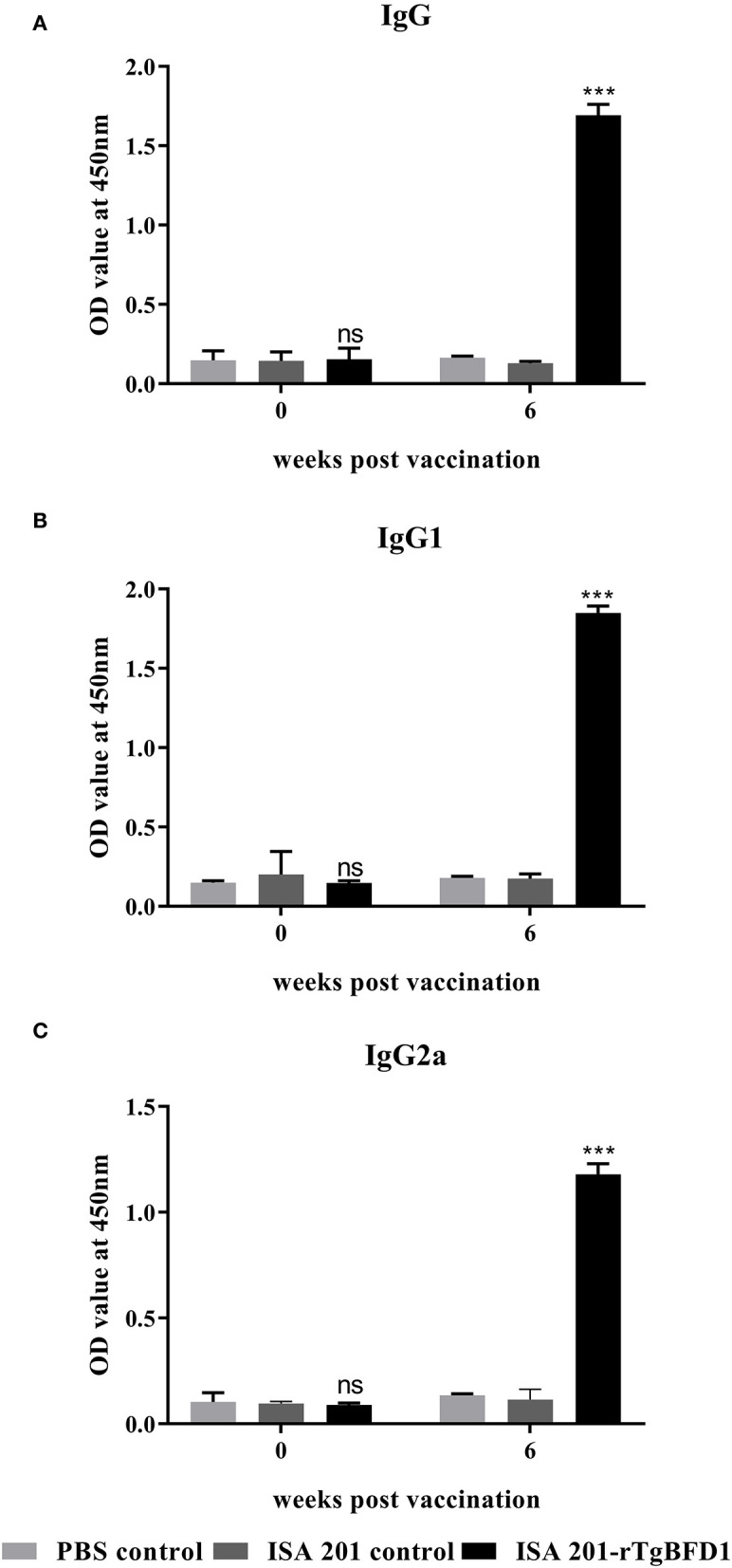
Specific antibody responses analyses in mice inoculated with rTgBFD1. **(A)** Alterations in sera-specific IgG contents at 0 and 6 weeks after vaccination. **(B)** Alterations in sera-specific IgG1 contents at 0 and 6 weeks after vaccination. **(C)** Alterations in sera-specific IgG2a contents at 0 and 6 weeks after vaccination. Data were shown as mean ± SD from ten mice per group and analyzed by a one-way ANOVA. ***p < 0.001 and ns (non-significant) compared with the PBS control and the ISA 201 adjuvant group.

For detection of specific antibody response, mice sera were collected after 6 weeks post-immunization and tested by ELISA against rTgBFD1 proteins. As illustrated in Figure 3, mice that received rTgBFD1 inoculations reached anti-rTgBFD1 titers of 1:10^4^. In Figure 4, mice sera antibody levels did not differ significantly between all groups before vaccination (*p* > 0.05). After three immunizations, sera-specific IgG contents against rTgBFD1 appeared a significant increase (*p* < 0.001, respectively) compared with PBS control and ISA 201 adjuvant group (Figure 4A). Similarly, the same considerable rise tendencies were observed both in the IgG1 and IgG2a levels after vaccinations (*p* < 0.001, respectively), and IgG1 was the major subtype (Figures 4B,C).

### Cytokine response to rTgBFD1 vaccination

**Figure 5:**
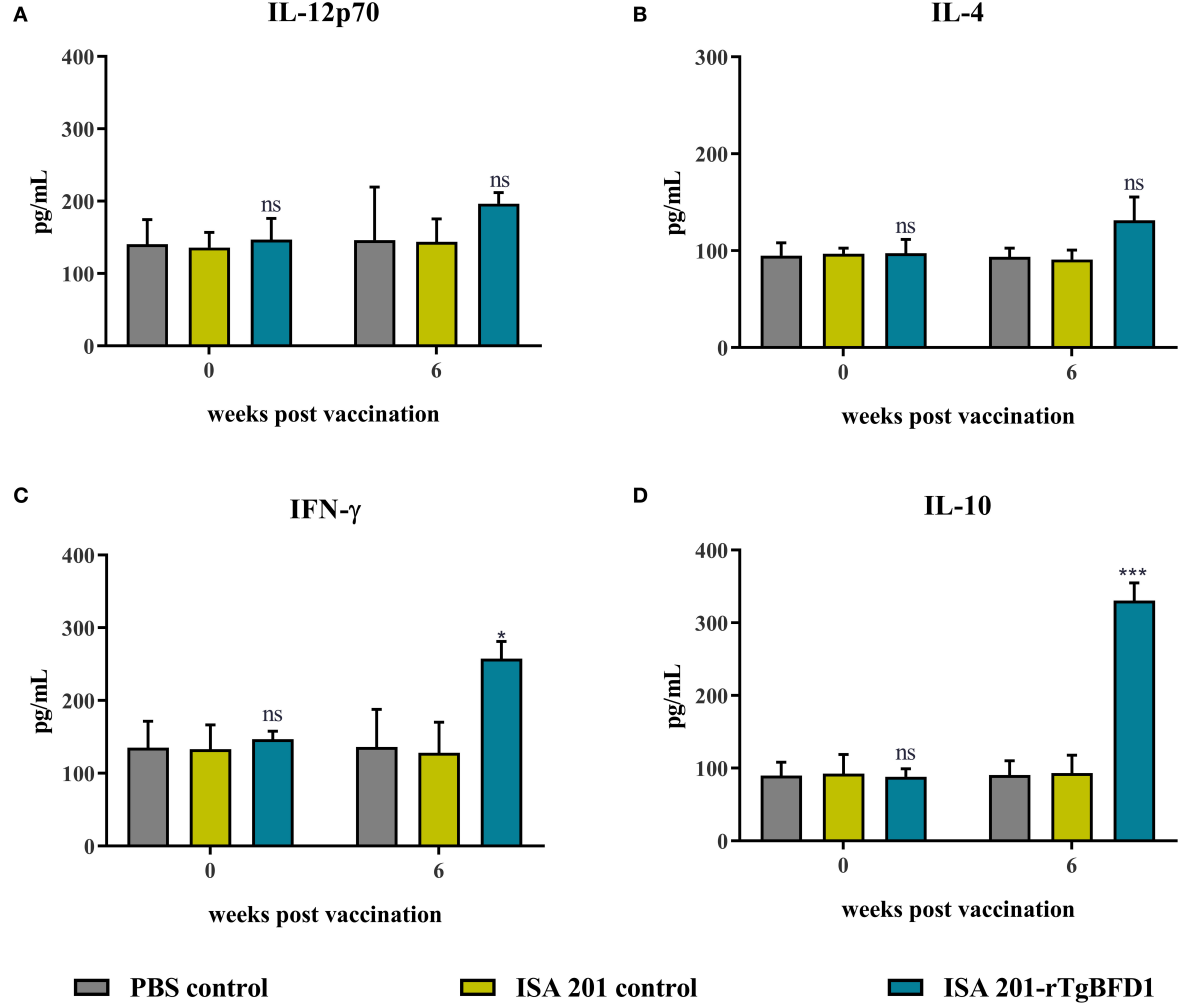
Multiple cytokines IL-12p70 **(A)**, IL-4 **(B)**, IFN-γ **(C)**, IL-10 **(D)** concentrations of rTgBFD1 vaccinated mice at 0- and 6-weeks post-immunization. Data were shown as mean ± SEM from ten mice per group and analyzed by a one-way ANOVA. *p < 0.05, ***p < 0.001 and ns compared with the PBS control and the ISA 201 adjuvant group.

To determine cellular immune response induced by TgBFD1, serum cytokine levels (IL-12p70, IL-4, IFN-γ, and IL-10) were detected using ELISA. Serum samples were obtained before immunization (week 0) and at 6 weeks post-immunization with ISA 201-rTgBFD1. As observed in Figure 5, mice sera multiple cytokines levels did not differ significantly between all groups before vaccination (*p* > 0.05). Approximately 6 weeks after immunization, there were no statistical differences in IL-12p70 and IL-4 contents among the groups (*p* > 0.05, respectively) (Figures 5A,B). By contrast, IFN-γ and IL-10 levels in ISA 201-rTgBFD1 immunized mice sera were notably higher than that in control groups (*p* < 0.05 and *p* < 0.001, respectively) (Figures 5C,D).

### Immunization with rTgBFD1 was effective against *T. gondii* infection

**Figure 6:**
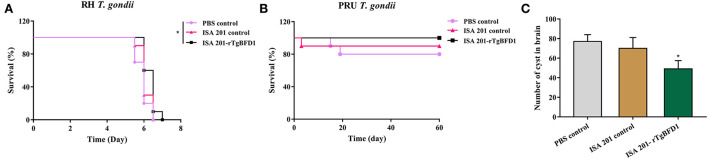
Mice survival time analyses and brain cyst number statistics. **(A)** Mice survival time analyses in *T. gondii* RH strain infection assay. Data were analyzed by the Kaplan–Meier method. **(B)** Mice survival times analyses in the *T. gondii* PRU strain infection assay. The survival of PRU strain-infected mice was observed for a 60-day period. Data were analyzed by the Kaplan–Meier method. *p < 0.05 vs. PBS control group. **(C)** Brain cyst number statistics of mice challenged with the *T. gondii* PRU strain. Data were presented as mean ± SEM and analyzed by a one-way ANOVA. *p < 0.05 vs. PBS control group.

Intraperitoneal infection of 10^2^ tachyzoites of *T. gondii* RH strain led to all the mice's death around 7 days post infection (Figure 6A). By contrast, mice survival time did not present a significant difference between PBS control and ISA 201 adjuvant groups (*p* > 0.05). However, in ISA 201-rTgBFD1 immunized group, mice survival time was monitored slightly longer than the PBS control group (*p* < 0.05).

As indicated in the Figure 6B, all mice from the ISA 201-rTgBFD1 immunized group infected oral gavage with 10 cysts of *T. gondii* PRU strain survived over 2 months of observation. However, there were one mouse in the ISA 201 adjuvant group and two mice in PBS control group died in this assay. In particular, of primary concern was the brain cyst burdens, which decreased markedly in ISA 201-rTgBFD1 immunized mice brains compared with the PBS control group (*p* < 0.05, Figure 6C), with a rate of 36.4% (Table 1).

**Table 1:** The number of PRU cysts in rTgBFD1 pre-immunized mice brain.

**Groups**	**Cysts number in brain (mean ±SEM)**	**Cysts reduction rate (%)**
sPBS control	77 ± 7^a^	0.00
ISA 201 control	70 ± 11.1^ab^	9.09
ISA 201-rTgBFD1	49 ± 8.6^b^	36.4

Same letters in same column represent non-significant difference, *p* > 0.05. Different letters in same column represent significant difference, *p* < 0.05.

## Discussion

Focusing on the current and potential harms posed by *T. gondii*, toxoplasmosis vaccine development for humans is required. Immunization with a live attenuated vaccine might lead to more excellent immunogenicity. However, as compared to this approach, recombinant subunit vaccines had exceptional safety and were rare to suffer any side effects since they were composed of high purity antigens. Thus, the identification of specific target proteins was the most essential factor for such a platform to work efficiently (23). In this study, the antigen TgBFD1 exhibited promising immunogenicity in the BALB/c mouse models. However, the immunization strategy entailing the use of adjuvant ISA 201 to improve the protective efficacy of rTgBFD1 was not satisfactory and improved adjuvant selection, or a more available antigen delivery system should be considered. Besides, according to the assessment of TgBFD1 vaccine efficacy, ISA 201-rTgBFD1 emulsion performed better in chronic toxoplasmosis infection than that in mice acute infection. This observation deserves more attention, so we believed that TgBFD1 still existed theoretical possibility to be a vaccine candidate.

The highly antigenic proteins are presumed as the most appropriate vaccine candidates (24). In the present study, we found that TgBFD1 has an important advantage owing to its immunogenicity, which is indicated by the epitope analysis. Further investigations also provided compelling evidence about the immunogenicity of rTgBFD1. In terms of Western blot and ELISA assays, TgBFD1 immunization not only elicited a strong rTgBFD1-specific humoral immune response but also could be identified by *T. gondii* infected mice sera. Therefore, it is amply documented that TgBFD1 (921-1023aa) selected in this study was immunogenic.

As is known, *T. gondii* is an intracellular parasitic protozoan, Th1 immune response induced by the vaccine is advantageous for the host (13). The analysis of specific immune responses generated by rTgBFD1 suggested that ISA 201-rTgBFD1 can elicit both cell and humoral immunity with a mixed Th1/Th2 cells response. The elevated specific IgG, IgG1, and IgG2a contents indicated that rTgBFD1 triggered an IgG1-dominant immune response (Th2), which might lead to a very limited contribution to the survival of immunized mice challenged with *T. gondii*. Besides, we also attempted to elucidate the immunologic mechanisms from the changes in cytokine contents perspective. As the Th1-type cytokines, IL-12 and IFN-γ have significant roles in preventing *T. gondii* infection (25). As seen in published studies, IFN-γ could induce immunity against toxoplasmosis by effectively facilitating CD8^+^ T cell maturation to exert its cytotoxic functions (26, 27). Meanwhile, IL-12 was able to serve a synergistic effect during the infection stage. The concentration of cytokine IL-12 did not have elevation trend, but as expected, IFN-γ contents exhibited a clear ascending trend compared with controls, which might help to prevent *T. gondii* infection and to promote cysts clearance from mice brains. As a key immunoregulator, cytokine IL-10 could inhibit Th1 cell activities to maintain a balanced immune response (28) and played significant roles in controlling inflammatory responses throughout the *T. gondii* acute infection period (29, 30). Thus, as observed in IL-10 levels, an upward trend was presented at 6 weeks post-immunization compared with controls. It is well known that IL-4 as a Th2 cytokine would normally exhibit the antagonistic function of IFN-γ (31). The detection results of IL-4 concentrations appeared insignificant changes in the present study.

For the reasons of *T. gondii* virulence and infection route, two strains (*T. gondii* RH and PRU) were selected for our research. To date, a complete protection against the *T. gondii* RH strain, which displayed a highly virulent in mice strains, is not available. After rTgBFD1 vaccination, limited protection mediated by the recombinant proteins was observed in the acute assay. Although the rTgBFD1 inoculations prolonged the survival time of mice from the point of view of statistical analysis, there was an extra average of 0.5 days more than the mice survival time in the PBS control group. However, this does not mean that the rTgBFD1 vaccine has no value. Noteworthy is that the method of intraperitoneally infecting *T. gondii* tachyzoites in mice commonly performed in experimental studies was due to its facile operation. However, this manner was not a natural route. Therefore, oral inoculation with *T. gondii* cysts by mimicking a natural infecting manner was warranted (32). In the chronic assay, the *T. gondii* PRU cyst number investigated in mice immunized with rTgBFD1 suggested a significant degradation of cyst load, which might be realized by the specific antibodies and multiple cytokines induced by the vaccination of rTgBFD1. From the outcome of the animal assays, the rTgBFD1 vaccine had a better performance in the *T. gondii* PRU cyst infection stage. Furthermore, it is critical to recognize that only RH and PRU strains were verified in the current study, that different virulence of *T. gondii* strains should be taken into account, and whether rTgBFD1 could generate efficient protection against *T. gondii* required more investigations.

As we all know, the primary purpose of active vaccination is to develop protective immune responses against *T. gondii* infection. Given that the vaccines based on a single recombinant protein do not provide satisfactory protection after infection with *T. gondii*, multiple-immunogen combination vaccine should be considered when optimizing future vaccine regimens. In addition, some other factors may influence the effectivity of inoculation procedures (e.g., mice gender, breeds, age, vaccination plans, *T. gondii* strains as well as sources of *T. gondii* cysts, etc.) (33). Moreover, in view that the vaccination will induce remarkably complex immune responses in mice, we still lacked a systemically summary and comprehensive evaluation of the immune response produced by rTgBFD1 under several parameters in mice experiments. Hence, the optimization of vaccination programs and more comprehensive immune response detections generated by rTgBFD1 should be taken into consideration in further research. Only then can the satisfying vaccine candidate be attained.

## Conclusion

On the whole, this study suggests that TgBFD1 is a good immunogen. Subcutaneous boosting with rTgBFD1 emulsified with ISA 201 adjuvant not only contributes to a strong humoral and cellular immune response but also has better protective efficacy for the hosts against chronic *T. gondii* infection. However, in spite of some desirable properties indicating that rTgBFD1 can be studied as a vaccine candidate from *T. gondii* infected mouse models, further integrated investigations are still required to enhance the immune protective effects of TgBFD1.

## Data availability statement

The raw data supporting the conclusions of this article will be made available by the authors, without undue reservation.

## Ethics statement

The animal study was reviewed and approved by the Ethics Committee of Xinxiang Medical University (Approval number XYLL-2021S016).

## Author contributions

Conception or design of this study: XL, SW, and XT. Direction and supervision: XM, ZZ, and XL. Data analysis and drafting of this article: XT and ZY. Material acquisition: XT, GW, TX, HS, and MW. All authors read and approved the final manuscript.

## Funding

The current work received support from the Science and Technology Planning Project of Henan Province (Nos. 212102310749 and 222102310557), the Key Scientific Research Projects of Colleges and Universities in Henan Province (22A310004), the Doctoral Scientific Research Activation Foundation of Xinxiang Medical University (No. XYBSKYZZ202139), and the Scientific Research Cultivation Project of School of Basic Medical Sciences in Xinxiang Medical University (Nos. JCYXYKY202115 and JCYXYKY202103).

## Conflict of interest

The authors declare that the research was conducted in the absence of any commercial or financial relationships that could be construed as a potential conflict of interest.

## Publisher's note

All claims expressed in this article are solely those of the authors and do not necessarily represent those of their affiliated organizations, or those of the publisher, the editors and the reviewers. Any product that may be evaluated in this article, or claim that may be made by its manufacturer, is not guaranteed or endorsed by the publisher.
